# The effect of surface texture on the kinetic friction of a nanowire on a substrate

**DOI:** 10.1038/srep44907

**Published:** 2017-03-21

**Authors:** Hongtao Xie, James Mead, Shiliang Wang, Han Huang

**Affiliations:** 1 School of Mechanical and Mining Engineering, The University of Queensland, QLD4072, Australia

## Abstract

The friction between Al_2_O_3_ nanowires and silicon substrates of different surface textures was characterised by use of optical manipulation. It was found that surface textures had significant effect on both the friction and the effective contact area between a nanowire and a substrate. A genetic algorithm was developed to determine the effective contact area between the nanowire and the textured substrate. The frictional force was found to be nearly proportional to the effective contact area, regardless of width, depth, spacing and orientation of the surface textures. Interlocking caused by textured grooves was not observed in this study.

The nanoscale contact between two objects may generate extremely strong adhesion and hence significantly great friction, which would considerably hinder the mobility of the objects^1^,^2^,^3^. Kinetic nanofriction can affect the wear of moving components in a device, thus shortening the device life^4^. However, nanofriction could also be utilized for developing products such as dry adhesives^5^, and wall-climbing “StickyBot” robots^6^. It is well known that macroscale friction is induced by the elastic or elasto-plastic deformation of surface asperities according to the classical theory of mechanics^7^,^8^,^9^, and the friction thus increases with a rougher surface. At nanoscale, friction is, however, enhanced when surfaces become smoother as van der Waals (vdW) attraction plays a dominant role^10^,^11^,^12^,^13^,^14^,^15^. Consequently, roughening the contact surfaces is expected to reduce nanofriction^16^,^17^,^18^,^19^,^20^,^21^.

A great research effort has been directed towards understanding the effect of roughness on nanofriction in the past decades^10^,^11^,^12^,^22^,^23^. Nevertheless, few studies were concerned with the understanding of the role of surface texture^24^,^25^,^26^,^27^. Previous studies showed that the frictional force of a micro-scale ball-tip sliding on a surface with periodic microscale grooves was heavily dependent on their contact angle, which varied with the relative position of the ball center with respect to the groove or the ratio between ball diameter and groove width^25^,^26^,^27^. The friction between two laser-fabricated surfaces of nanoscale periodic grooves was found to be dependent on texture orientation. This suggested that geometric interlocking, i.e. the in-plane restriction of the motion between surface grooves and ridges, played a significant role in this case^19^.

Nanowires (NWs) have been recently used as moving components in nanodevices^28^,^29^. The understanding of the friction between a NW and its supporting substrate is thus critical in the design and development of the NW-based devices, and thus has attracted increasingly more attention of research in recent years^30^,^31^,^32^,^33^. The nanoscale friction is typically characterised by the frictional shear stress or the frictional force per unit contact area. The friction between a NW and a substrate was often measured using the direct measurement via atomic force microscopy (AFM)^32^,^34^,^35^ or the indirect way through measuring the bent shape of the NW^30^,^36^,^37^,^38^,^39^. Previous studies showed that the frictional shear stress at NW/substrate interfaces varied from several to several tens of mega-pascals^32^,^34^,^35^,^37^,^40^,^41^,^42^, which was influenced by the NW and substrate materials^41^,^42^,^43^. Nevertheless, those studies were performed on atomically smooth substrates. The effect of surface texture of the substrate on nanofriction has yet to be understood. It is thus essential to examine how surface texture affects the nanofriction and whether geometric interlocking occurs at NW/substrate interfaces.

In this study, we investigated the effect of surface texturing of a Si substrate on the friction of Al_2_O_3_ NWs through nanomanipulating. We thus developed a general genetic algorithm (GA) to calculate the contact area between a sliding NW and its supporting substrates of different surface textures. The kinetic friction forces and the contact areas on the textured and smooth surfaces were compared. The role of contact area in nanofriction was revealed and the underlying mechanism on the effect of surface texturing was discussed.

## Experimental details

**Figure 1:**
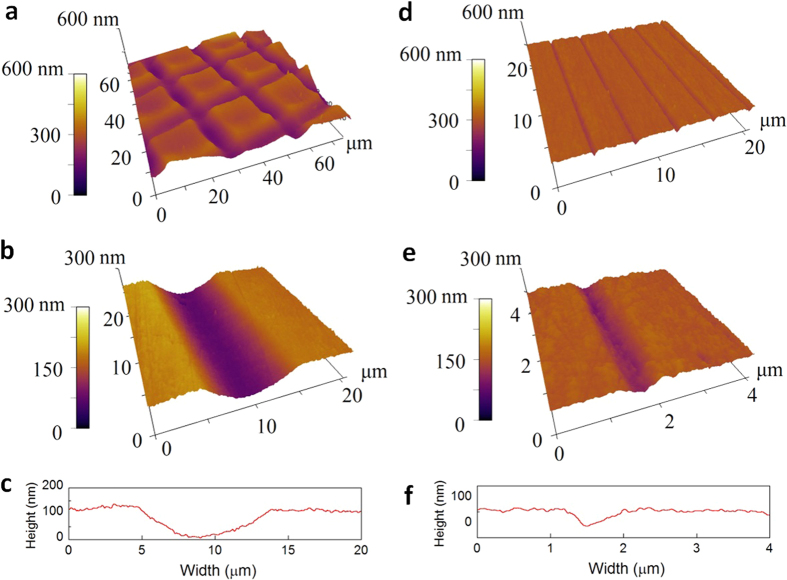
AFM images of the grooved Si wafers. (**a**) The grooved grid on a Si wafer with a spacing of 25 μm; (**b**) the three-dimensional image and (**c**) the corresponding two-dimensional profile of the groove in (**a**) shows with hg = 120 nm and *w*_*g*_ = 8 μm wide; (**d**) the parallel grooves with a spacing of *w*_*g*_ = 4 μm; (**d**) three-dimensional image and (**e**) the corresponding two-dimensional profile of the groove in (**f**) shows with *h*_*g*_ = 50 nm and *w*_*g*_ = 0.9 μm. Here *w*_*g*_, *h*_*g*_ and *s*_*g*_ are the width, depth and spacing of the grooves, respectively.

The as-received Al_2_O_3_ NWs have a rectangular cross-section and atomically smooth surface^44^. A commercially available single-crystalline Si wafers with an average roughness of 0.8 nm (obtained from a scanning area of 20 × 20 μm^2^) was textured in this study. The substrates were grooved to have different textures using nanoscratching performed on a HYSITRON Triboindenter^®^. Berkovich and conical diamond tips with tip radii of 100 nm and 100 μm, respectively, were used to produce narrow and broad grooves. After scratching, the substrates were mechanically cleaned using tightly woven cotton tips soaked with liquid ethanol, followed by ultrasonic cleaning, aiming at removing abrasion debris and producing the smoothest possible surface in the grooved area. A surface roughness of 2.1 nm was found within the grooves and the adjacent area near the grooves after mechanical cleaning. (Noted the roughness value was measured from a scanning area of 20 × 20 μm^2^ on the textured surface, using a high-pass 2RC filter^45^ with a wavelength of 0.5 μm). Figure 1 shows typical AFM images of the substrates textured. Figure 1a shows a grid pattern with an average groove width of 8 μm and spacing of 25 μm, which was produced using the conical tip. The high magnification AFM image in Fig. 1b and the profile shown in Fig. 1c show that the grooves have a sinusoidal-like cross-sectional profile, which is 120 nm deep. Figure 1e shows a texture pattern of parallel grooves that have an average width of 900 nm and a spacing of 4 μm. The high-magnification image in Fig. 1e and the profile in Fig. 1f shows that the grooves have a cross-sectional profile of 50 nm in depth. Twenty-two different surface textures were tested in this work, and the texture patterns are summarised in Table 1.

The friction of a NW sliding on a textured substrate was measured by use of the optical nanomanipulation technique developed in our previous works^33^,^41^,^44^. During testing, each NW was pushed at its centre using a tungsten tip, so it slid on the substrate at a constant speed. On each surface texture, six NWs were used to in the sliding test. The testing processes were monitored by optical microscopy (Objective lens: Mitutoyo M Plan APO 50× and HR100×) at a temperature of ~25 °C and relative humidity of ~45%. The dimensions and sizes of the NWs and substrates surface textures were examined by AFM (Asylum Research MFP-3D) and confocal scanning microscopy (Lext OLS4100).

## Results and discussion

**Figure 2:**
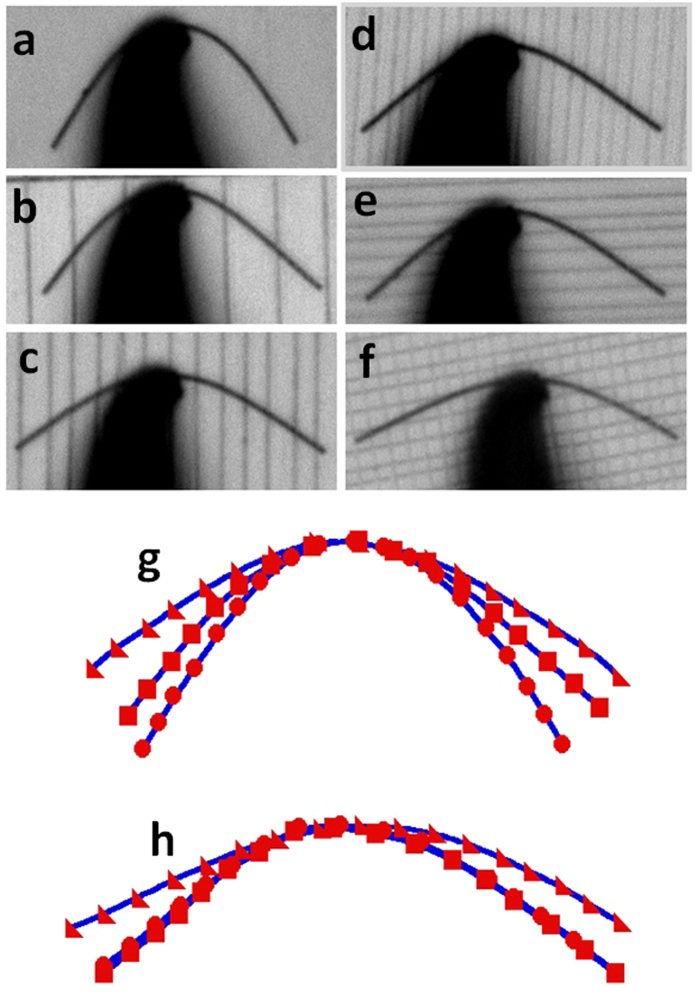
Optical images of an Al_2_O_3_ NW being pushed to slide on (**a**) the smooth Si surface with a roughness of 2.1 nm; (**b**) the textured Si surfaces along the direction parallel to the grooves with *w*_*g*_ = 0.9 μm, *h*_*g*_ = 5 nm and *s*_*g*_ = 6 μm, (**c**) *w*_*g*_ = 0.9 μm, *h*_*g*_ = 5 nm, and *s*_*g*_ = 3 μm, (**d**) *w*_*g*_ = 0.7 μm, *h*_*g*_ = 30 nm, and *s*_*g*_ = 2 μm; (**e**) the textured Si surfaces along the direction perpendicular to the grooves with *w*_*g*_ = 0.7 μm, *h*_*g*_ = 30 nm, and *s*_*g*_ = 2 μm, (**f**) Grids with grooves of *w*_*g*_ = 0.7 μm, *h*_*g*_ = 3  nm and *s*_*g*_ = 2 μm. (**g**) The skeletonized NW shapes, where circle, square and triangle represents the NW in (**a**–**c**), respectively. (**h**) The skeletonized NW shapes, where circle, square and triangle represents the NW in (**d**–**f**) respectively. Here *w*_*g*_, *h*_*g*_ and *s*_*g*_ are the width, depth and spacing of the grooves, respectively.

Figure 2 shows the optical images of the bent NW sliding on the textured substrates. Using model of non-linear beam subjected to uniformly distributed load, the friction at the NW/substrate interface is calculated as^33^,


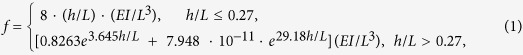


where *f* is the kinetic friction per unit length, *L* is the length of the NW measured from one end to the centre point*, h* is the distance from the centre of NW to the line connecting the two ends of the NW measured from the optical image. *E* and *I* are the elastic modulus and the second moment of area of the NW, respectively.

In Fig. 2a,c, the same NW was pushed to slide on the three substrates of different textures. Figure 2a shows the smooth wafer surface of roughness of 2.1 nm, Fig. 2b shows the surface with parallel grooves of *w*_*g*_ = 0.9 μm, *h*_*g*_ = 50 nm, and *s*_*g*_ = 6 μm, and Fig. 2c shows the surface with parallel grooves of *w*_*g*_ = 0.9 μm, *h*_*g*_ = 50 nm, and *s*_*g*_ = 3 μm. Here *w*_*g*_, *h*_*g*_ and *s*_*g*_ are the width, depth and spacing of the grooves, respectively. Figure 2d,f show the bending profiles of the same NW on the substrates with sliding directions perpendicular, parallel to the grooves and over the grid pattern, respectively. In all these three textures, the grooves have the same values of *w*_*g*_ = 0.7 μm, *D* = 30 nm and *s*_*g*_ = 2.0 μm. It can be seen that the bent profiles are not significantly affected by the groove orientation. Figure 2g,h show the skeletonized NW shapes in Fig. 2a–c and d–f, respectively. According to the NW profiles, the friction force is not significantly dependent on the groove direction, but clearly decreases with an increasing density of grooves.

**Figure 3:**
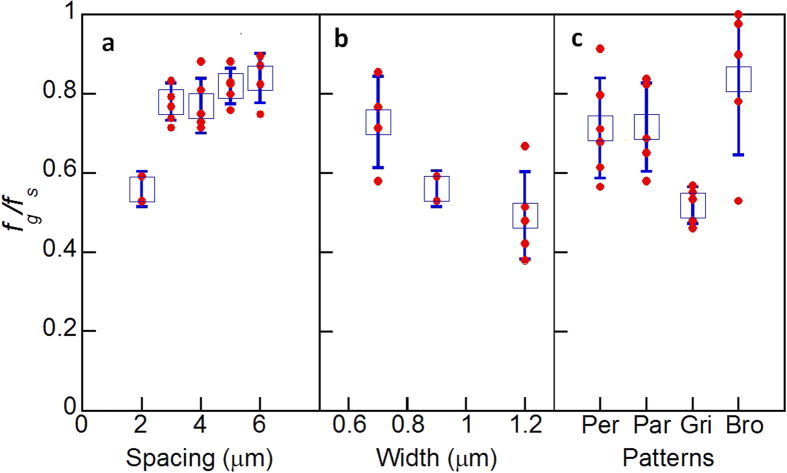
The normalized friction force per unit length, *f*_*g*_/*f*_*s*_, plotted as a function of groove (**a**) spacing, (**b**) width and (**c**) orientation. In (**c**), Per and Par stand for sliding perpendicular to parallel to the groove length direction; while Gri means the sliding on the grid texture and Bro represents the sliding on the broad grid. Note that red dots represent experimental data, blue blocks are the average values and the error bars are the standard derivation.

To understand the effect of surface texture on the friction of NWs, comparative tests were carried out using the texture patterns shown in Table 1. Figure 3 shows the results obtained from the tests. It should be noted in Fig. 3 that the frictional force of a NW sliding on a textured surface was normalised using the value measured on the smooth surface, thus giving a non-dimensional parameter, *f*_*g*_/*f*_*s*_.where *f*_*g*_ and *f*_*s*_ are the kinetic friction per unit length for the NW on the grooved substrate and smooth substrate, respectively. As shown in Fig. 3a, an increase in groove spacing resulted in the increased frictional force, but the increasing rate with the smaller spacing was much more substantial. It is clearly seen in Fig. 3b that *f*_*g*_/*f*_*s*_ decreased with the increased groove width. Figure 3c shows the effect of different texture patterns. Sliding in a direction parallel or perpendicular to the grooves provided similar friction force results. However, sliding on the surface of a grid texture, which can be considered the superposition of both parallel and perpendicular groove textures, is much smaller. Surprisingly, the friction measured from sliding on a grid of broad grooves is the greatest among the four textures.

**Figure 4:**
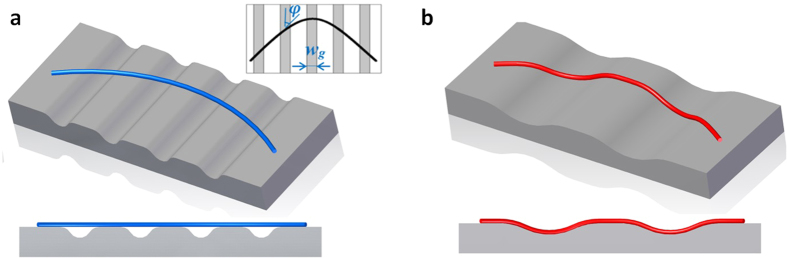
Schematic illustration of a NW (**a**) span over narrow grooves, and (**b**) in contact with the bottom of broad grooves.

Previous studies have shown that the frictional force of a NW on a smooth substrate is dependent on the contact area^36^,^46^. A question arises is if the rule applies to the sliding of a NW on a texture surface. To find out this, the contact area between a textured surface and a NW must be measured, which is quite difficult as NWs are often flexible and grooves on the textured surface are extremely shallow. Basically, two contact scenarios may appear. As shown in Fig. 4a, the sliding NW might span over narrow grooves on the surface without contact with the grooves. However, in Fig. 4b the NW would conform to the profile of broad grooves due to relatively strong vdW attraction. (See AFM images in the Supplementary Figure S2) This could lead to a significant difference in the determination of contact area. A quantitative criterion based on the classic theory of elasticity could be used to predict the contact status between a NW and a grooved surface. Assuming that the cross-sectional profile of the surface grooves is sinusoidal, the criterion can be written as, (see more details in Appendix 1)


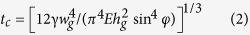


where *t*_*c*_ is the critical thickness of the NW, γ is interface energy for the NW/substrate system, *φ* is the angle between a tangent on the longitudinal axis of the bent NW and the groove direction, and *E* is the elastic modulus of the NW. For a NW with a thickness of *t*, if *t* > *t*_*c*_, the NW will span over the groove, and if *t* > *t*_*c*_, the NW will be in contact with the bottom of the grooves. Substituting the typical parameters of the narrow grooves and NWs used in the tests, *w*_*g*_ = 1.2 μm, *h*_*g*_ = 60 , *φ* = *π*/4, *E* = 310 nm GPa^47^ and γ = 1 mJ/m^2^ (see Appendix 1) into Equation (2), we obtained *t*_*c*_ ≈ 10 nm, which is significantly smaller than the NW thickness used in our tests. This indicates that the NWs being tested would be unable to conform to the grooves during sliding. Additionally, the surface profile shown in Fig. 1f appears not as gradual as a sinusoidal wave. This means that a larger elastic energy would be required to conform a NW to the actual groove profile. In this case, the effective contact area *A* for the NW/substrate interface can be simply estimated by,


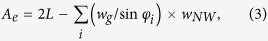


where 2*L* is the total length of the NW, *w*_*NM*_ is the NW width.

**Figure 5:**
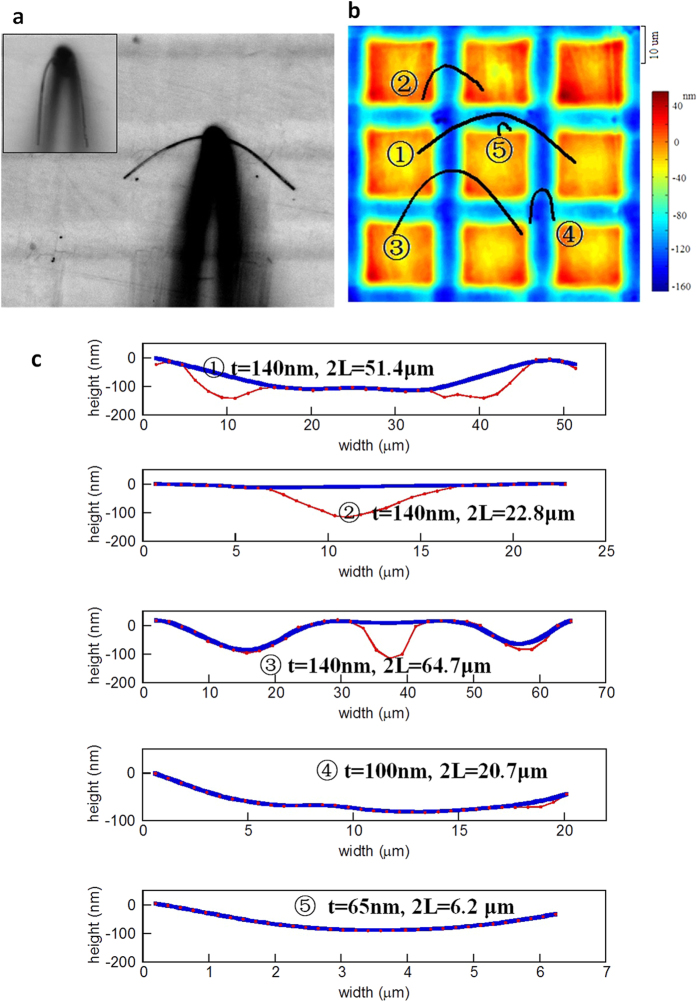
(**a**) An Al_2_O_3_ NW sliding on a Si surface with broad grooved grid pattern of *w*_*g*_ = 8 μm, *h*_*g*_ = 120 nm, and *s*_*g*_ = 25 μm. The inset shows the same NW sliding on the smooth substrate (**b**) The skeletonized shapes of 5 different NWs sliding on different locations of the grooved substrate shown in (**a**), where 1 represents the shape of the NW in (**a**). (**c**) The cross-sectional profiles of different contact statuses of NW 1–5 shown in (**c**). Red dotted lines represent the cross-sectional substrate profiles underneath the NWs, and the blue solid curves are the NW profiles calculated from the genetic algorithm.

When sliding on the surface with broad grooves (Fig. 5a), a NW may partially or completely conform to the profile of the grooves, due to the relatively great groove width, *w*_*g*_ and great ratio of width over height, *w*_*g*_/*h*_*g*_. Figure 5b shows the confocal microscopic image of the grid with the skeletonized profiles of five NWs sliding on the surface, where NW 1 represents profile of the sliding NW shown in Fig. 5a. The thicknesses of the NWs, labelled as no. 1 to 5 in Fig. 5b, are 140, 140, 140, 100 and 65 nm, respectively. Substituting the characteristic values into Equation (2) with *w*_*g*_ = 8 μm, *h*_*g*_ = 120 nm and *φ* = *π*/4, we obtain *t*_*c*_ ≈ 76 nm, which is close to the NW thicknesses used. This suggests that Equation (3) is no longer suitable to calculate the contact area for the surfaces of broad grooves. Using the 2D interpolation function in MatLab, the surface profiles of the substrate underneath the five NWs can be extracted from Fig. 5b, which were plotted as the red curves in Fig. 5c. Apparently, the profiles cannot be simply assumed as sinusoidal. To estimate the effective contact area, *A*_*e*_ between a NW and the substrate with broad grooves, we developed a novel genetic algorithm based on the lowest energy principles (see Appendix 2 for details). The contact profile between a NW and the substrate was thus able to be derived using the genetic algorithm and then plotted as the blue solid curves in Fig. 5c. It is seen that NW 1, 3, 4 and 5 are in contact with the side wall and bottom of the grooves and NW 2 spans across the groove.

**Figure 6:**
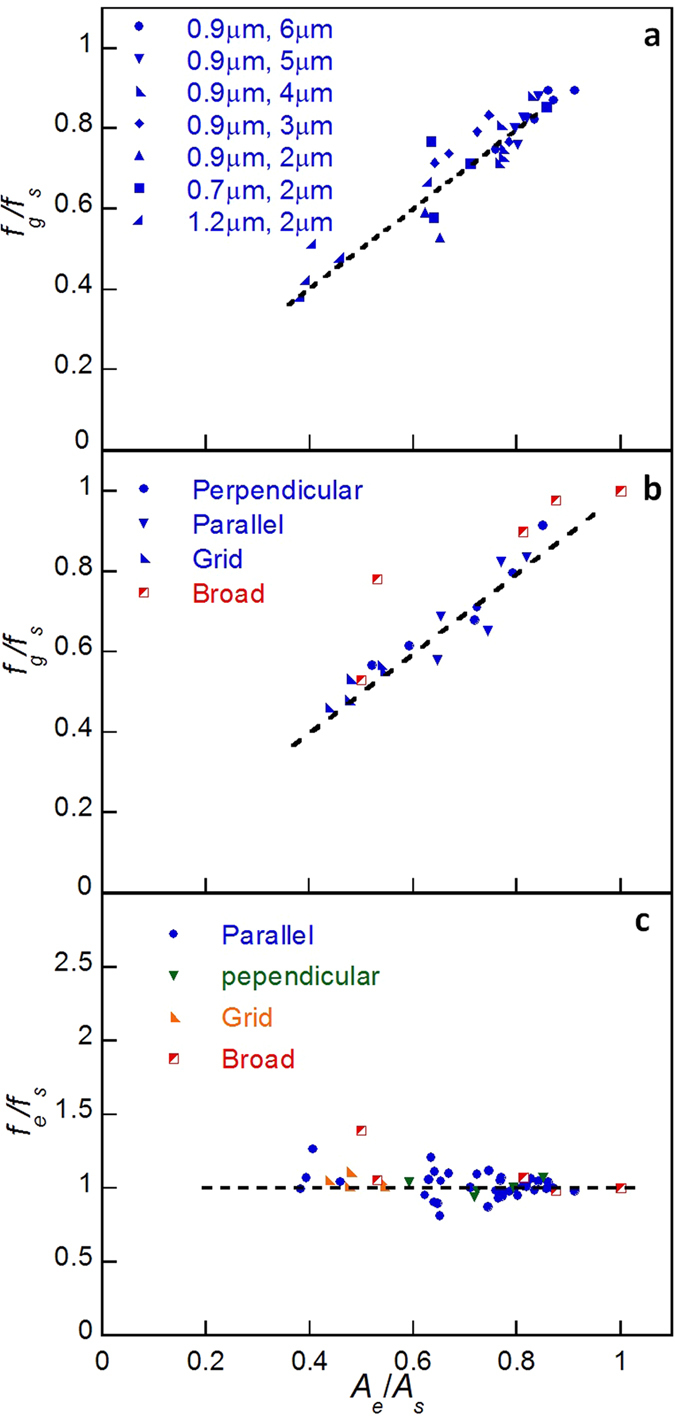
(**a**) Normalized friction per unit length plotted as a function of normalized contact area for Si substrates textured with parallel grooves of different widths and spacings. (**b**) Normalized friction per unit length plotted as a function of normalized contact area for different textured surfaces with the same groove width and spacing. (**c**) Frictional force per unit effective contact length, *f*_*e*_ normalized by *f*_*s*_ verses *A*_*e*_/*A*_*s*_.

The effective contact areas for NWs on all the textured surfaces listed in Table 1 were obtained by applying Equation (3) or the genetic algorithm on their corresponding surface groove type. The frictional force data in Fig. 3 is replotted in Fig. 6a,b as a function of normalised effective contact area, *A*_*e*_/*A*_*s*_. Note that the normalising parameter, *A*_*s*_, is the contact area of a NW on a smooth surface. For all narrow groove textured surfaces, the normalised friction force follows an almost linear relationship with the normalised contact area. However, for the texture with grooves of 8 μm wide, *f*_*g*_/*f*_*s*_ somehow deviates from the linear relationship. This is because the frictional force can no longer be considered uniformly distributed along the length of the NW when the width of the broad grooves is close to the length of the NW. Under this circumstance, the frictional force per unit effective contact length, *f*_*e*_, should be used, rather than *f*_*g*_.

**Figure 7:**
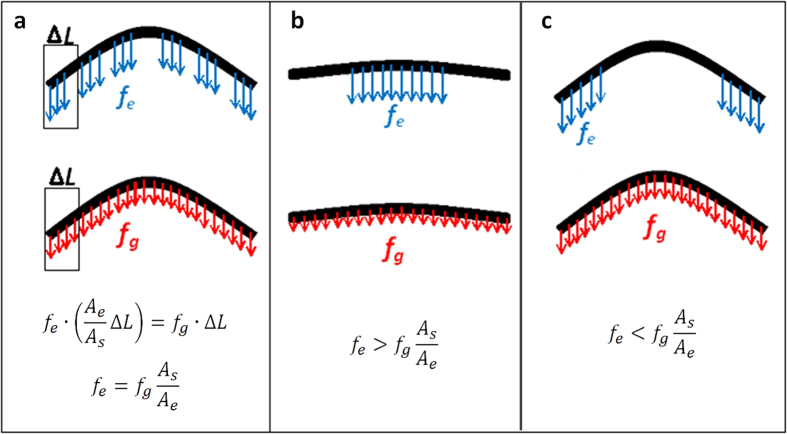
Schematic illustrations of frictional force distributions for the contacts between a NW and textured surfaces with (**a**) narrow grooves, (**b**) broad grooves with contact at the centre of the NW and (**c**) broad grooves with contacts at the ends of the NW.

As shown in Fig. 7a, when a NW slides on a surface with narrow grooves, the friction force acting on the NW is composed of small discrete force segments uniformly distributed along the length direction. Therefore, *f*_*e*_ can be simply calculated as *f*_*g*_*A*_*s*_/*A*_*e*_. However, when a NW slides on a surface with broad grooves, the frictional force is composed of a few large force segments, which cannot be treated as the uniformly distributed force along the NW, as exampled in Fig. 7b,c. When the friction force acts at the centre of the NW (see Fig. 7b), using *f*_*g*_*A*_*s*_/*A*_*e*_ to represent *f*_*e*_ underestimates the friction force; while the friction force is overestimated if it acts at the ends of the NW (see Fig. 7c). As a consequence, when sliding on the surface with broad grooves the frictional force per unit effective length must be determined by considering the effect of such non-uniform distribution. In this study, finite element modelling (FEM) was used to estimate *f*_*e*_. FEM models were established using ANSYS for the cases with non-uniform force distributions. An initial nodal force was applied onto the nodes along the NW where contact with substrate was identified. The nodal force was then iteratively adjusted until the best fit between the simulated and experimental bent profiles was achieved. The nodal force being achieved was considered as the friction force per node, so the frictional force per unit effective contact length, *f*_*e*_, was determined. Figure 6(c) shows the normalized frictional force per unit effective contact length, *f*_*e*_/*f*_*s*_, plotted against the normalised effective contact area, *A*_*e*_/*A*_*s*_, for all the surface textures. It is seen that the frictional forces per unit effective contact length on a textured surface in fact equals that on a smooth surface, regardless of groove width, spacing and orientation. This also suggests that interlocking did have insignificant effect on the frictional stress, even though in some cases the groove width is much greater than the NW diameter. This result is different from those reported previously^19^,^24^,^25^,^26^,^27^, where interlocking appears having played an important role in determining the friction. In our tests it is likely that the NWs used might be insufficiently short to cause interlocking. The atomically smooth surfaces of NWs would certainly reduce the possibility of interlocking too. Nevertheless, we expect that the extremely short NWs would be trapped on relatively broad and deep grooves. The phenomenon that a NW can conform onto a textured surface is quite similar to the conformation of 2D nanomaterials, such as graphene films, onto a rough substrate, which is considered as the main cause for friction enhancement and thickness-dependent friction^48^,^49^,^50^. The genetic algorithm developed in this study could potentially be extended to calculate the contact statues between 2D nanomaterials and the underlying substrate, and thus improve the understanding of friction of 2D nanomaterials. Our study also suggests that by changing the texture of substrate texture the friction of a nanostructure on a rigid substrate could be tailored. There are some uncertainties that require attention in the measurement. First, the frictional force might not be uniformly distributed along the NW because of the discrete distribution of texture grooves. In this case, the contact point between the tip and the NW would be slightly off the centre to achieve the balance. The error caused by the tip position was expected to lead to an uncertainty of 5% in measuring L, which in turn resulted in an uncertainty of approximately 15% in friction estimation using Equation (1). Second, contamination or wear of NWs or substrates could also affect the friction in theory. However, in our study such effects would not significantly alter the results. This is because the friction stress in magnitude of a few MPa in our test was insufficiently strong to generate significant wear. Both the surfaces of NW and substrate were reasonably clean, so the contamination wouldn’t significantly change the friction value, either. Third, some burrs were formed at the edges of grooves, which could reduce the effective contact area and thus overestimate the friction calculated by Equation (3).

## Conclusions

The effect of surface texture on the friction of the NWs on Si substrates was systematically investigated. A NW could span across relatively narrow grooves, but might be in contact with the bottom of relatively broad grooves, dependent on the adhesion energy and elastic compliance of the NW. When a NW spanned across the grooves, the frictional force decreased with the width and density of the grooves. When the NW conforms to the relatively broad grooves, a genetic algorithm was developed to determine the effective contact area. For both the narrow or broad surface textures being studied in this work, the frictional force per unit length of a NW was nearly proportional to the effective contact area, regardless of groove width, spacing or orientation. Our study clearly indicated that the effect of substrate texture on the frictional force of a NW was dominantly through the change in its contact area with the substrate surface. This finding sheds light on the understanding of the friction between one-dimensional nanostructures and their underlying substrates, which is extremely valuable for the applications of one-dimensional nanostructures into nanodevices.

## Additional Information

**How to cite this article:** Xie, H. *et al*. The effect of surface texture on the kinetic friction of a nanowire on a substrate. *Sci. Rep.* **7**, 44907; doi: 10.1038/srep44907 (2017).

**Publisher's note:** Springer Nature remains neutral with regard to jurisdictional claims in published maps and institutional affiliations.

## Supplementary Material

Supplementary Information

## Figures and Tables

**Table 1:** Geometric parameters of substrate texture patterns.

Orientation	Width (μm)	Spacing (μm)	Depth (nm)	Tip
parallel	0.7	2	30	Berkovich
	0.9	2	50	
	1.2	2	60	
	0.9	3	50	
	0.9	4	50	
	0.9	5	50	
	0.9	6	50	
perpendicular	0.7	2	30	
	0.9	2	50	
	1.2	2	50	
	0.9	3	50	
	0.9	4	50	
	0.9	5	50	
	0.9	6	50	
grid	0.7	2	30	
	0.9	2	50	
	1.2	2	60	
	0.9	3	50	
	0.9	4	50	
	0.9	5	50	
	0.9	6	50	
	8	25	120	Conical
