# Supplementary material for: The effect of surface texture on the kinetic friction of a nanowire on a substrate
Source: Sci Rep. 2017 Mar 21;7:44907. doi: 10.1038/srep44907 (PMC5359617; doi:10.1038/srep44907)
Supplement: Supplementary Information [file srep44907-s1.pdf]

Supplementary information

The effect of surface texture on the kinetic friction of a nanowire on a substrate

Hongtao Xie, James Mead, Shiliang Wang*, Han Huang*
School of Mechanical and Mining Engineering, The University of Queensland, QLD4072, Australia. E-mail: shiliang.wang@uq.edu.au; han.huang@uq.edu.au

Appendix 1. Criteria for a NW to completely contact with a sinusoidal groove

The cross-sectional profile of a groove is assumed to be a sinusoidal function,

$$z = \frac{h_g}{2}\cos\left(\frac{2\pi x \sin\varphi}{w_g}\right), \qquad (A1.1)$$

where x and z are lateral and normal axis coordinates, h_g and w_g are height and width of the groove, respectively. When the NW is fully conformed to the profile of the groove, the normal deflection shape of the NW is the same as Equation $(A1.1)$, As x is much larger than z, a linear approximation can be applied and the curvature function, k, of the NW can be expressed by,

$$k = \frac{d^2z}{dx^2} = \frac{-2\pi^2 h_g \sin^2\varphi}{w_g^2}\cos\left(\frac{2\pi x \sin\varphi}{w_g}\right). \qquad (A1.2)$$

The elastic energy, U_e, from the deformation of the NW in the normal direction is determined by,

$$U_e = \int_0^L \frac{EI}{2}k^2 \mathrm{ds} = \frac{\pi^4 E h_g^2 \sin^4\varphi \, w_{NW} t^3}{12 w_g^4} L, \qquad (A1.3)$$

where L, E and I are length, elastic modulus and second moment of area of the NW, respectively. As the NW is fully conformed to the groove profile, the elastic energy stored in the NW should be less than or equal to the interface energy between the NW and substrate, i.e.,

$$U_e \le \gamma w_{NW} L, \qquad (A1.4)$$

where γ is the surface energy per unit area, and can be estimated by, $\gamma = \sqrt{A_{NW}A_s}/[12\pi(d+d_{co})^2]$, [1] where $d = 2.1$ nm is the surface roughness and $d_{co} = 0.2$ nm is the cut-off distance, A_{NW} = 15.2 $\times 10^{-20}$ J [2] and A_s = 25.5 $\times 10^{-20}$ J [3] are the Hamaker constants for the NW and substrate materials, respectively. Thus, γ is estimated to be ~ 1 mJ/m^2 for the NW/substrate interface used in this study.

From Equations $(A1.3)$ and $(A1.4)$, we obtain the critical thickness, t_c

$$t_c \le \left(\frac{12\gamma w_g^4}{\pi^4 E h_g^2 \sin^4\varphi}\right)^{1/3}. \qquad (A1.5)$$

Appendix 2. Determining the contact area between a NW and an irregular surface using a genetic algorithm

Assuming that both a NW and its respective substrate can be discretised by n points along the NWs axial direction, the number of the possible contact status between the NW and substrate is thus 2^n. Physically, the real contact state for the NW/substrate system should correspond to the lowest total energy of the system, $E_{total} = U_e - \gamma w_{NW} L_{contact}$. Where $L_{contact}$ is the effective contact length at the NW/substrate interface.

Finding the best solution among 2^n possibilities is an non-polynomial (NP) problem, [4] and a genetic algorithm (GA) is frequently applied for practical applications.[5] A GA begins from a population of randomly generated individuals (solutions that usually represented by binary arrays or so called "chromosomes"), and then mimic the natural selection process to find the optimal individual after successive generations.[5]

Discrete points on the NW and substrate were noted as $(S_i, Z_{NW\,i})$ and $(S_i, Z_{ST\,i})$, respectively, where s_i is the curvature length from the *i*th point to the first point, and z_{NW} and z_{st} are the normal coordinate values of the NW and substrate profiles, respectively. A group of n-digit binary chromosomes (a sequence of 1's and 0's) representing the contact states (1 refers to contact, and 0 refers to non-contact) was randomly generated. This serves as the first generation (the initial population). The population size is the total number of chromosomes. Once these chromosomes were generated, the corresponding shapes of the NW can be determined in the following 2 steps:

First, at the *i*th point where the NW and substrate are in contact,

$$Z_{NW\,i} = Z_{ST\,i} \qquad (A2.1)$$

Second, the non-contact points of the NW were calculated using spline interpolation. Note that this is a simplification, as the NW is, in actuality, subjected to a normal vdW force on non-contact points. However, such deflection is negligible compared to the deflection resulting from contact with the substrate.

In each generation, the individual chromosomes can be ranked by a fitness function, defined as,

$$F = I - U_e - P, \qquad (A2.2)$$

where I, P and U are the interface energy, penetration penalty and elastic energy related to the NW deformation normal to the substrate surface, respectively. The interface energy, S, can be estimated by,

$$I = \Delta s w_{NW} \sum_{i=1}^{n} \sqrt{A_{NW} A_s} / [12\pi (Z_{NW\,i} - Z_{ST\,i} + d + d_{co})^2], \qquad (A2.3)$$

where Δs is the distance between two adjacent discrete points. The penalty for penetration P is defined as,

$$P = \sum_{i=1}^{n} (Z_{NW\,i} - Z_{ST\,i}) p_i \qquad (A2.4)$$

p_i is the pre-set penalty factor equals to a constant p when $Z_{NW\,i} - Z_{ST\,i} < 0$ and equals to zero when $Z_{NW\,i} - Z_{ST\,i} > 0$. p is negative and it is chosen to make P several magnitudes larger than U and I .This enables the algorithm to avoid selecting solutions where NW penetrates the substrate.

Practically, the genetic algorithm is developed and run in Matlab. Initially, the substrate profile were discretised into 33 points, and then 500 randomly generated 33-digit binary chromosomes served as the first generation population. The fitness function value, F, for these 500 individual chromosomes was ranked. The individual with a higher fitness function value will be assigned a larger possibility to “mate”, i.e. pass their genes to the next generation. The selected mating individuals will exchange some randomly chosen sections of their chromosome. After mating, the binary code of some randomly chosen individuals can mutate with a mutation rate of 0.05. In our model, the evolution process iterates through 500 generations in order to find the best solutions, as plotted in Figure 5 (c).

References

1. Houston, M. R., Howe, R. T. & Maboudian, R. Effect of hydrogen termination on the work of adhesion between rough polycrystalline silicon surfaces. *J. Appl. Phys*. **81**, 3474 (1997).
2. Bergstrom, L. Hamaker constants of inorganic materials. *Adv Colloid Interfac* **70**, 125-169 (1997).
3. Reinhardt, K. & Kern, W. *Handbook of silicon wafer cleaning technology* (William Andrew, 2008).
4. Cook, S. A. In *Proceedings of the third annual ACM symposium on Theory of computing*, 151–158 (ACM, Shaker Heights, Ohio, USA, 1971).
5. Mitchell, M. *An Introduction to Genetic Algorithms* (MIT Press, 1998).

Supplementary Figures

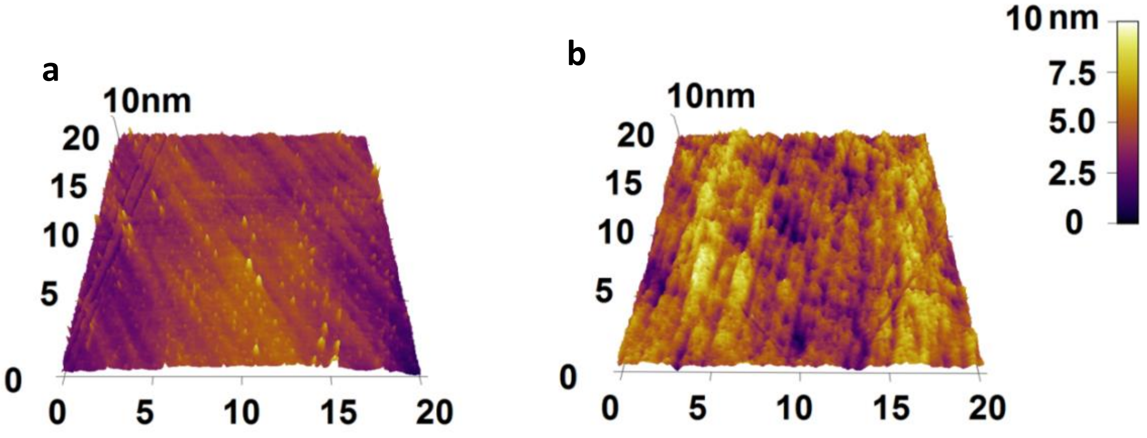

Figure S1. (a) The as-received Si substrate with an average roughness of 0.8 nm, and (b) the adjacent flat area near the grooves with an average roughness of 2.1 nm after mechanical cleaning

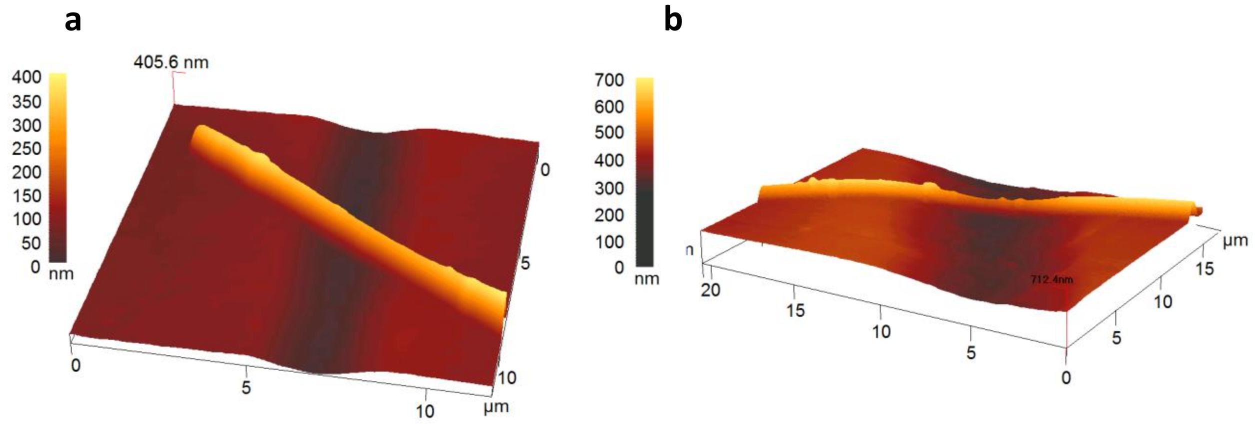

Figure S2: AFM images of a NW (a) spanning across a narrow groove, and (b) being in contact with the bottom of a broad groove.
